# Disclosing the benefits of multi-strain compounds and their health impact mechanisms utilizing intestinal biomimetic technology

**DOI:** 10.3389/fmicb.2025.1550913

**Published:** 2025-05-15

**Authors:** Jiahao Liao, Jianyu Huang, Xiaoqiong Li, Jian Kuang, Jianqiang Li, Weiwei Wang, Jinjun Li

**Affiliations:** ^1^College of Animal Science, Shanxi Agricultural University, Taigu, China; ^2^State Key Laboratory for Managing Biotic and Chemical Threats to the Quality and Safety of Agro-Products, Institute of Food Science, Zhejiang Academy of Agricultural Sciences, Hangzhou, China; ^3^Shanxi Key Laboratory of Animal Genetics Resource Utilization and Breeding, Taigu, China

**Keywords:** composite probiotics, *in vitro* culture, organoids, intestinal barrier, anti-inflammatory

## Abstract

The gut microbiota plays a vital function in preserving intestinal homeostasis and general health. This study examined the impacts of a composite probiotic based on an *in vitro* fermentation system and an organoid interaction research platform to investigate the effects of composite probiotics. 4P, investigating the impact of gut barrier function through the utilization of *in vitro* and organoid models. Our findings revealed that 4P demonstrated excellent capabilities for producing short-chain fatty acids, modulating intestinal gas metabolism, and regulating oxidative-reduction potential. Furthermore, the individual strains that comprise 4P were selected for subsequent organoid studies based on previous *in vitro* strain culture experiments. Our results suggest that 4P and its individual strains hold promise for preserving the gut barrier integrity and function, thereby potentially offering therapeutic benefits in the context of intestinal disorders. These findings underscore the importance of exploring the mechanistic actions of composite probiotics in maintaining gut health and preventing diseases.

## Graphical abstract

Effect of compound probiotics combination on maintaining intestinal health and preventing disease.
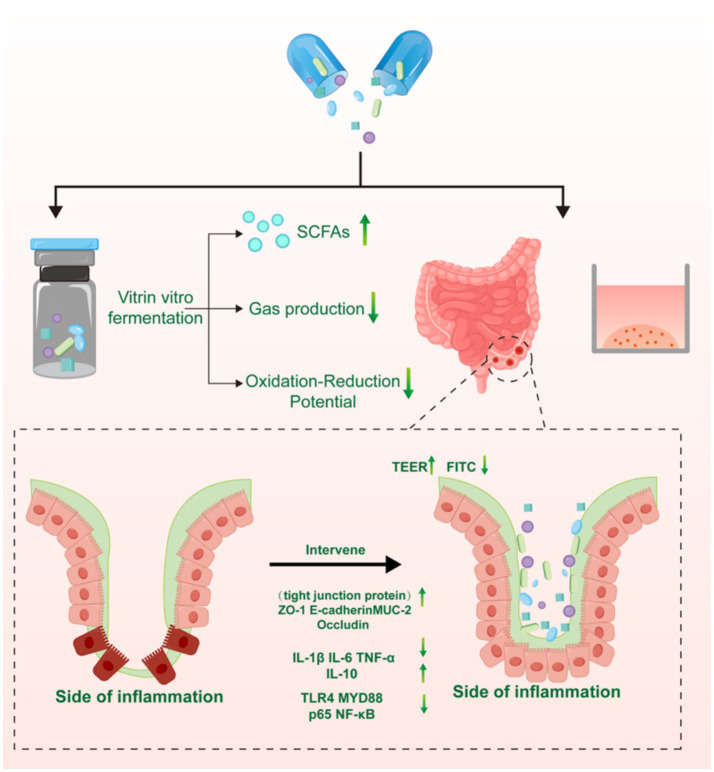

## 1 Introduction

The intestine, which is the most crucial immune organ, plays a crucial function in host-immune regulation, and interactions between intestinal microbiota are crucial. Prior research has suggested that the abundance of gut bacteria has a substantial effect in intestinal immune activation (Chen et al., 2024). The intestinal barrier protects the host from invasion by pathogens, viruses, or toxins. Diminished expression of intercellular tight junction proteins can result in decreased integrity of the intestinal barrier and heightened permeability, also known as “leaky gut.” This facilitates the penetration of pathogens through the intestinal barrier into the body, promoting intestinal inflammation and resulting in the dysregulation of intestinal immunity and intestinal microbiota (Liu et al., 2024; Yao et al., 2024). Therefore, the treatment of intestinal inflammation should focus on regulating intestinal microbiota and restoring intestinal function. Additionally, in recent research, strategies for the treatments of enteritis are increasingly moving towards safety and health, leading to widespread attention to the use of healthy probiotics (Stofilova et al., 2022; Murali and Mansell, 2024).

In this study, we adapted and optimized an *in vitro* gut fermentation system and an organoid interaction platform to better model gut microbial growth and epithelial interactions. This emerging approach is designed to simulate the gut microenvironment and its interactions with cells and tissues. The platform combines *in vitro* fermentation systems with organoid culture technology. Through *in vitro* fermentation, it simulates the growth of microbial strains in the gut, enabling the study of how probiotics alter the gut microenvironment and impact health. Organoids, which are small three-dimensional structures derived from stem cells, mimic the function and cellular composition of real organs. In gut research, intestinal organoids replicate the structure and function of the gut epithelium, allowing for deeper exploration of the effects of probiotics on gut function. This system provides researchers with a more precise means to investigate how gut microbes affect host cell health, revealing pathological mechanisms such as gut inflammation and metabolic imbalances, and aiding in drug screening and disease treatment research (Wu et al., 2020).

Probiotic strains can modulate the intestinal immune responses, enhance anti-inflammatory capabilities, regulate immune reactions, and exert immunostimulatory effects. Multiple studies have demonstrated the potential effectiveness of probiotics as a therapeutic approach for controlling immunological responses in the intestines and restoring the function of the intestinal barrier. For instance, *Bifidobacterium infantis* inhibits intestinal inflammation by suppressing the host toll-like receptor 4/nuclear factor kappa B (TLR4/NF-κB) and mitogen-activated protein kinase signaling pathways (Yue et al., 2023). In tumor necrosis factor-gamma (TNF-*γ*)-induced intestinal inflammation, *Lactobacillus reuteri* regulates intestinal immune function Through the activation of the Wnt/*β*-catenin signaling pathway, the healing of intestinal barrier damage is facilitated (Wu et al., 2020). Another study demonstrated that *Lactobacillus rhamnosus* can treat intestinal inflammation in mice with colitis by promoting the production of substances that reduce inflammation while decreasing the production of substances that promote inflammation (Fan et al., 2021). *Lactobacillus acidophilus* Modulates intestinal inflammation via influencing the equilibrium of regulatory T cells/T helper cells (Treg/Th17) (Xu et al., 2024). As the combination of *fragile Pseudomonas* and *Lactobacillus increases*, the body enhances cellular immunity by boosting the number of T cells in the spleen and mesenteric lymph nodes of mice (Naveed et al., 2024). *Bacillus subtilis* mitigates damage to the intestinal epithelial barrier by increasing the expression of tight junction proteins and ameliorating intestinal inflammation through the TLR4-NF-κB-NLRP3 inflammasome signaling pathway (Sheng et al., 2021). *Bacillus clausii* enhances the expression of tight junction proteins and treats intestinal damage by regulating the imbalance between pro-inflammatory and anti-inflammatory cytokines in the colon (Pirozzi et al., 2023). *Bacillus amyloliquefaciens* controls the growth and specialization of intestinal stem cells through the aryl hydrocarbon receptor/signal transducer and activator of transcription 3 pathway, thereby alleviating lipopolysaccharide-induced intestinal inflammation in piglets (Wang et al., 2024). With increasing research on probiotics, multi-strain probiotic formulations have gradually gained preference. Multi-strain probiotics exhibit better anti-inflammatory and barrier repair functions than single-strain probiotics (He et al., 2023; Han et al., 2023). Furthermore, multi-strain probiotics can create the environmental conditions necessary for the survival of probiotic strains. The presence of interactions among strains also allows mutual influence, resulting in different and superior roles and functions compared to those of individual strains (Zhou et al., 2024). Additional research is needed to examine the impact of novel probiotic combinations on intestinal inflammation and barrier function.

According to previous studies, the NF-κB transcription factor family is essential for regulating cellular inflammatory responses and immunological reactions. It is also a major regulatory factor of epithelial integrity and an effective target for the treatment of intestinal inflammation (Giri et al., 2022). TNF-*α* and TLR ligands are known activators of NF-κB signaling transduction. NF-κB activation has been detected in macrophages in colonic mucosal samples obtained from patients with colitis. Furthermore, in primary epithelial cell cultures derived from individuals diagnosed with inflammatory bowel disease (IBD), the chemokine interleukin (IL)-8 is significantly upregulated and regulated by NF-κB (Mukherjee et al., 2024; Bai et al., 2023).

This study employed an *in vitro* model to assess the potential benefits of a quadruple combination of probiotics on intestinal inflammation and barrier repair. The quadruple combination of probiotics included lactobacilli, bifidobacteria, streptococci, and *Bacillus* species, all of which have been shown to modulate intestinal inflammation and barrier function. To gain a clear understanding of the impact of the quadruple combination probiotic on intestinal inflammation and the intestinal barrier, we conducted comparative studies on short-chain fatty acids (SCFAs), gas production, gas types, and the redox potential of different combinations of probiotics. In studies on intestinal inflammation and the intestinal barrier, intestinal organoid models outperform traditional two-dimensional epithelial cell and animal models. Intestinal organoid models can present as three dimensional structures and better simulate the physiological structure and function of the intestinal epithelium *in vitro* (Tan et al., 2023). We initiated an inflammatory model in intestinal organoids by administering IFN-*γ* and assessed gene expression at several stages of the NF-κB pathway, encompassing receptors and downstream signaling molecules, along with pro-inflammatory cytokines and tight junction proteins linked to the intestinal barrier. Previous studies have demonstrated that the quadruple combination probiotic (4P) exhibits significant short-term efficacy in treating diarrhea-predominant irritable bowel syndrome (IBS), suggesting the potential of 4P in promoting gut health. However, the underlying mechanisms of 4P in regulating intestinal inflammation and protecting the intestinal barrier remain unclear. Therefore, our study aims to investigate its mechanisms of action in maintaining gut health.

## 2 Results

### 2.1 Metabolism of SCFAs by probiotics in different treatment groups

**Figure 1:**
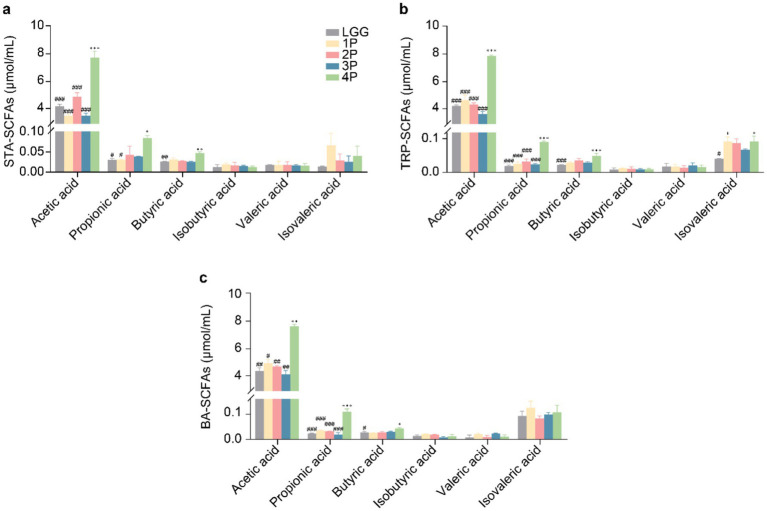
Comparison of SCFAs production among LGG and different groups of probiotics. **(a)** SCFAs under STA culture conditions; **(b)** SCFAs under TRP culture conditions; **(c)** SCFAs under BA culture conditions. Data are presented as mean ± SEM, analyzed using two-way ANOVA test. * vs. LGG, # vs. 4P; * *p* < 0.05, ** *p* < 0.01, *** *p* < 0.001; # *p* < 0.05, ## *p* < 0.01, ### *p* < 0.001.

In starch (STA) culture medium (Figure 1a), the four-strain combination probiotics (4P) group exhibited significantly higher levels of acetic acid than the other groups (*p* < 0.001). Propionic acid content was significantly higher in the 4P group than in the *Lactobacillus rhamnosus* GG (LGG) and single strain (1P) groups (*p* < 0.05), whereas butyric acid content was significantly higher in the 4P group than in the LGG group (*p* < 0.01). In tryptophan (TRP) culture medium (Figure 1b), acetic and propionic acid levels were significantly higher in the 4P group than in the other groups (*p* < 0.001), butyric acid content was significantly higher in the LGG group than in the 4P group (*p* < 0.001), and isobutyric acid content was significantly higher in the 4P and 1P groups than in the LGG group (*p* < 0.05). In bile acid (BA) culture medium (Figure 1c), acetic and propionic acid levels were significantly higher in the 4P group than in the other groups (*p* < 0.05), whereas butyric acid levels were significantly higher in the LGG group than in the other groups (*p* < 0.05).

### 2.2 Gas metabolism and redox potential of probiotics in different treatment groups

**Figure 2:**
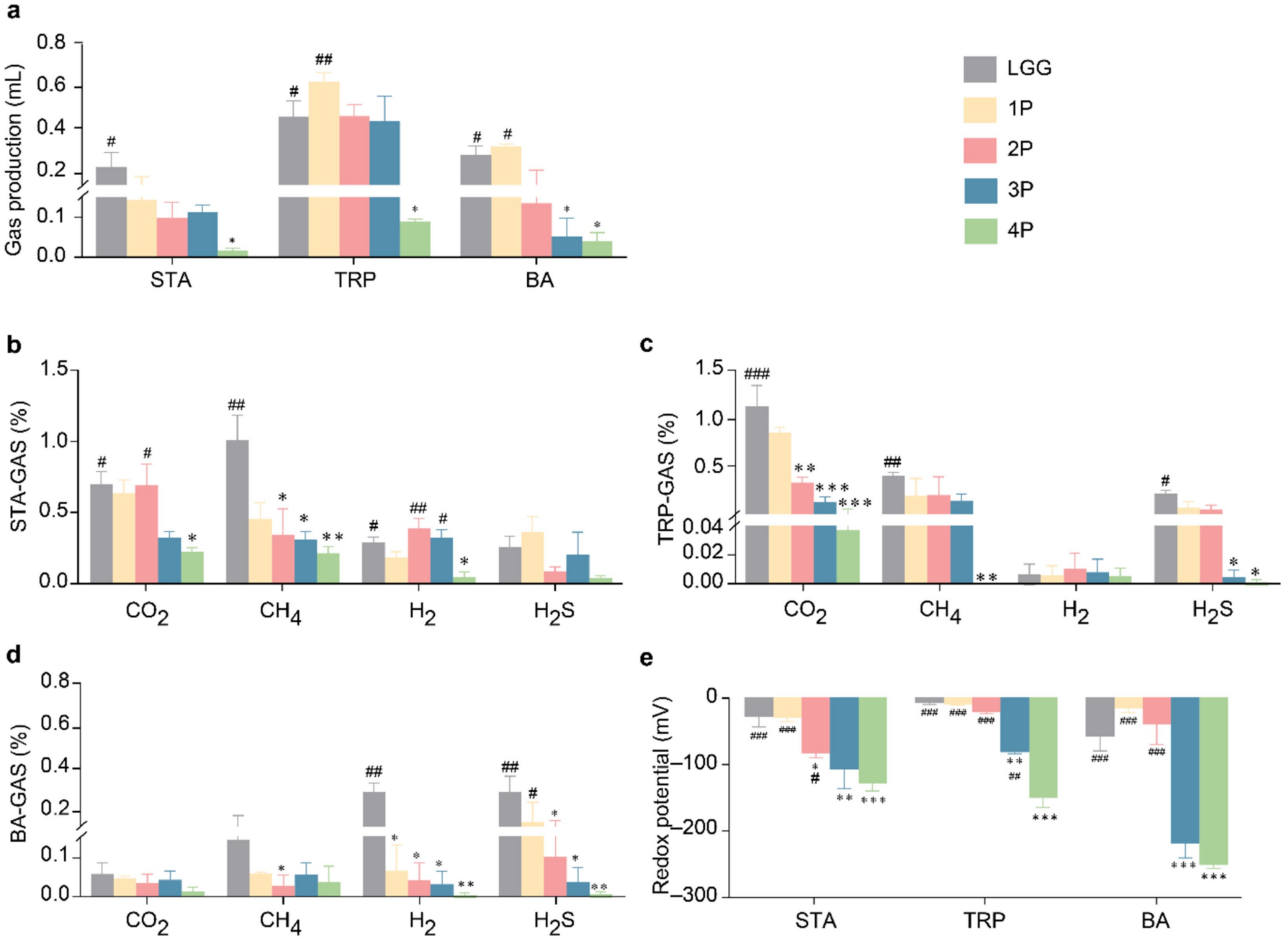
Comparison of gas metabolism and redox potential among LGG and different groups of probiotics. **(a)** Gas production under STA, TRP, and BA culture conditions; **(b)** Gas metabolism under STA culture conditions; **(c)** Gas metabolism under TRP culture conditions; **(d)** Gas metabolism under BA culture conditions; **(e)** Redox potential under STA, TRP, and BA culture conditions. Data are presented as mean ± SEM, analyzed using two-way ANOVA test. * vs. LGG, # vs. 4P; * *p* < 0.05, ** *p* < 0.01, *** *p* < 0.001; # *p* < 0.05, ## *p* < 0.01, ### *p* < 0.001.

In terms of gas production (Figure 2a) in STA culture medium, gas production in the 4P group was significantly lower than that in the LGG group (*p* < 0.05). In TRP culture medium, gas production in the 4P group was significantly lower than that in the LGG and 1P groups (*p* < 0.05). In BA culture medium, gas production in the 4P and three-strain combination probiotics (3P) groups was significantly lower than that in the LGG and 1P groups (*p* < 0.05). In STA culture medium (Figure 2b), CO_2_ production in the 4P group was significantly lower than that in the LGG and two-strain combination probiotics (2P) groups (*p* < 0.05). CH_4_ production in the 4P, 3P, and 2P groups was significantly lower than that in the LGG group (*p* < 0.05). Additionally, H_2_ production in the 4P group was significantly lower than that in the LGG, 2P, and 3P groups (*p* < 0.05). In TRP culture medium (Figure 2c), CO_2_ production in the 4P, 3P, and 2P groups was significantly lower than that in the LGG group (*p* < 0.01). CH_4_ production in the 4P group was significantly lower than that in the LGG group (*p* < 0.01). H_2_S production in the 4P and 3P groups was significantly lower than that in the LGG group (*p* < 0.05). In BA culture medium (Figure 2d), CH_4_ production in the 2P group was significantly lower than that in the LGG group (*p* < 0.05). H_2_ production in the 4P, 3P, 2P, and 1P groups was significantly lower than that in the LGG group (*p* < 0.05). H_2_S production in the 3P and 2P groups was significantly lower than in the LGG group (*p* < 0.05), and H_2_S production in the 4P group was significantly lower than that in the 1P and LGG groups (*p* < 0.05).

Regarding the redox potential (Figure 2e), in STA medium, the redox potential in the 4P group was significantly lower than that in the LGG, 1P, and 2P groups (*p* < 0.05). The redox potentials in the 2P, 3P, and 4P groups were significantly lower than that in the LGG group (*p* < 0.05). In TRP medium, the redox potential in the 4P group was significantly lower than that in the other groups (*p* < 0.01), and the redox potential in the 3P group was significantly lower than that in the LGG group (*p* < 0.01). In BA medium, the redox potential in the 3P group was significantly lower than that in the LGG group (*p* < 0.001), and the redox potential in the 4P group was significantly lower than that in the LGG, 1P, and 2P groups (*p* < 0.001).

### 2.3 Metabolism of SCFAs by the different probiotics

Based on the results of previous studies, the composite probiotic 4P demonstrated excellent SCFA production, intestinal gas metabolism, and redox potential. Therefore, individual investigations were conducted on the four probiotic strains comprising the composite probiotic 4P.

**Figure 3:**
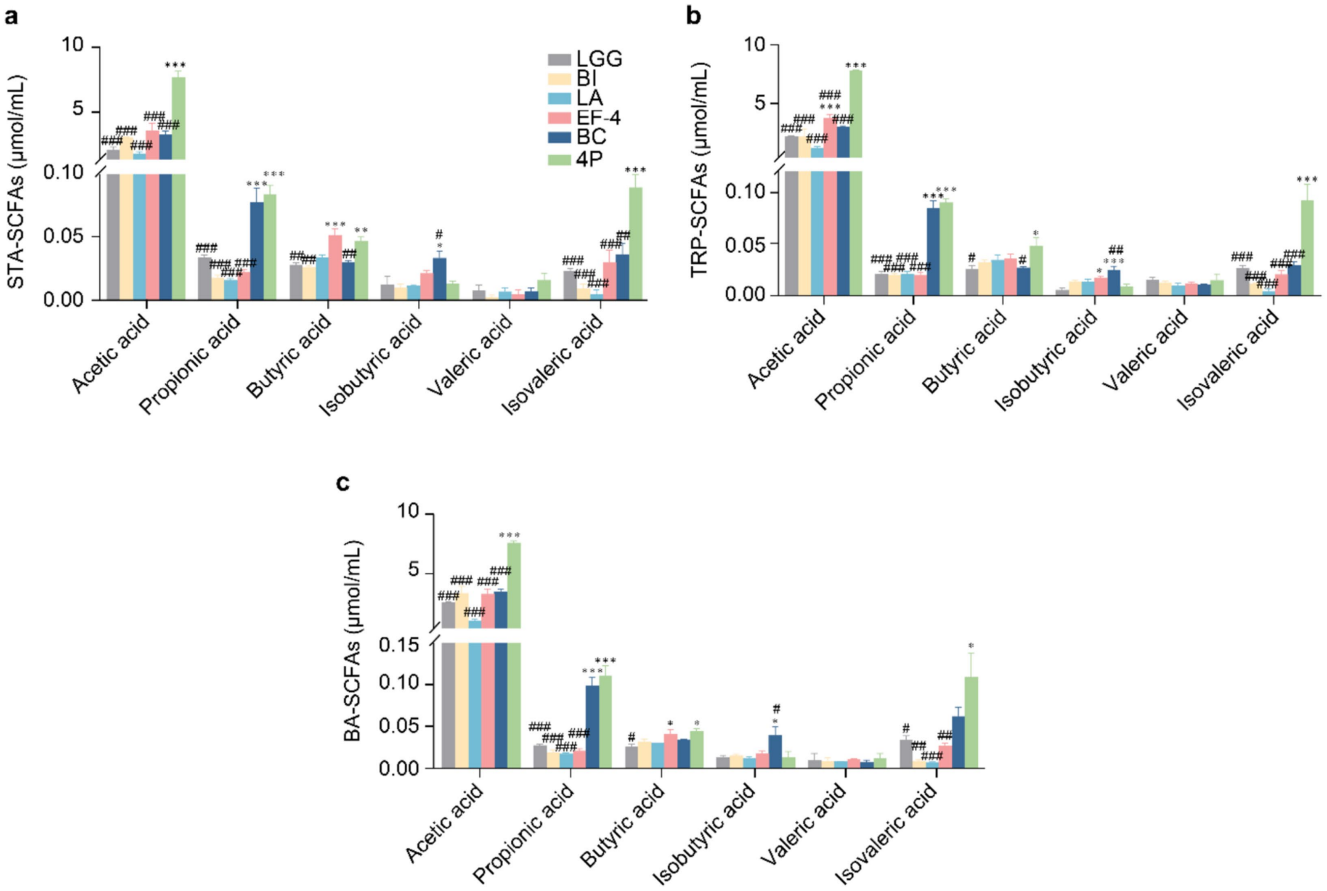
Comparison of SCFAs production among different probiotics. **(a)** SCFAs produced under STA culture conditions; **(b)** SCFAs produced under TRP culture conditions; **(c)** SCFAs produced under BA culture conditions. Data are presented as mean ± SEM, analyzed using a two-way ANOVA test. * vs. LGG, # vs. 4P; * *p* < 0.05, ** *p* < 0.01, *** *p* < 0.001; # *p* < 0.05, ## *p* < 0.01, ### *p* < 0.001.

In STA medium (Figure 3a), acetic and isovaleric acid levels in the 4P group were significantly higher than those in the individual strain groups (*p* < 0.01). Propionic acid levels in the 4P and *Bacillus cereus* (BC) groups were significantly higher than those in the other groups (*p* < 0.001). Butyric acid levels in the 4P and *Enterococcus faecalis*-4 (EF-4) groups were significantly higher than those in the other groups (*p* < 0.01), and the isobutyric acid level in the BC group was significantly higher than that in the LGG group (*p* < 0.05). In TRP medium (Figure 3b), acetic and isovaleric acid levels in the 4P group were significantly higher than those in the individual strain groups (*p* < 0.001). Propionic acid levels in the 4P and BC groups were significantly higher than those in the other groups (*p* < 0.001). Butyric acid production was significantly higher in the 4P group than that in the LGG and BC groups (*p* < 0.05). Isobutyric acid levels in the EF-4 and BC groups were significantly higher than that in the LGG group (*p* < 0.05). In BA medium (Figure 3c), the acetic acid level in the 4P group was significantly higher than that in the individual strain groups (*p* < 0.001). Propionic acid levels in the 4P and BC groups were significantly higher than those in the other strains (*p* < 0.001). Butyric acid levels in the 4P and EF-4 groups were significantly higher than that in the LGG group (*p* < 0.05). Isobutyric acid levels in the BC group were significantly higher than that in the LGG group (*p* < 0.05), and isovaleric acid levels in the 4P group were significantly higher than those in the LGG, *Bifidobacterium infantis* (BI), *Lactobacillus acidophilus* (LA), and EF-4 groups (*p* < 0.05).

### 2.4 Gas metabolism and redox potential of the different probiotics

**Figure 4:**
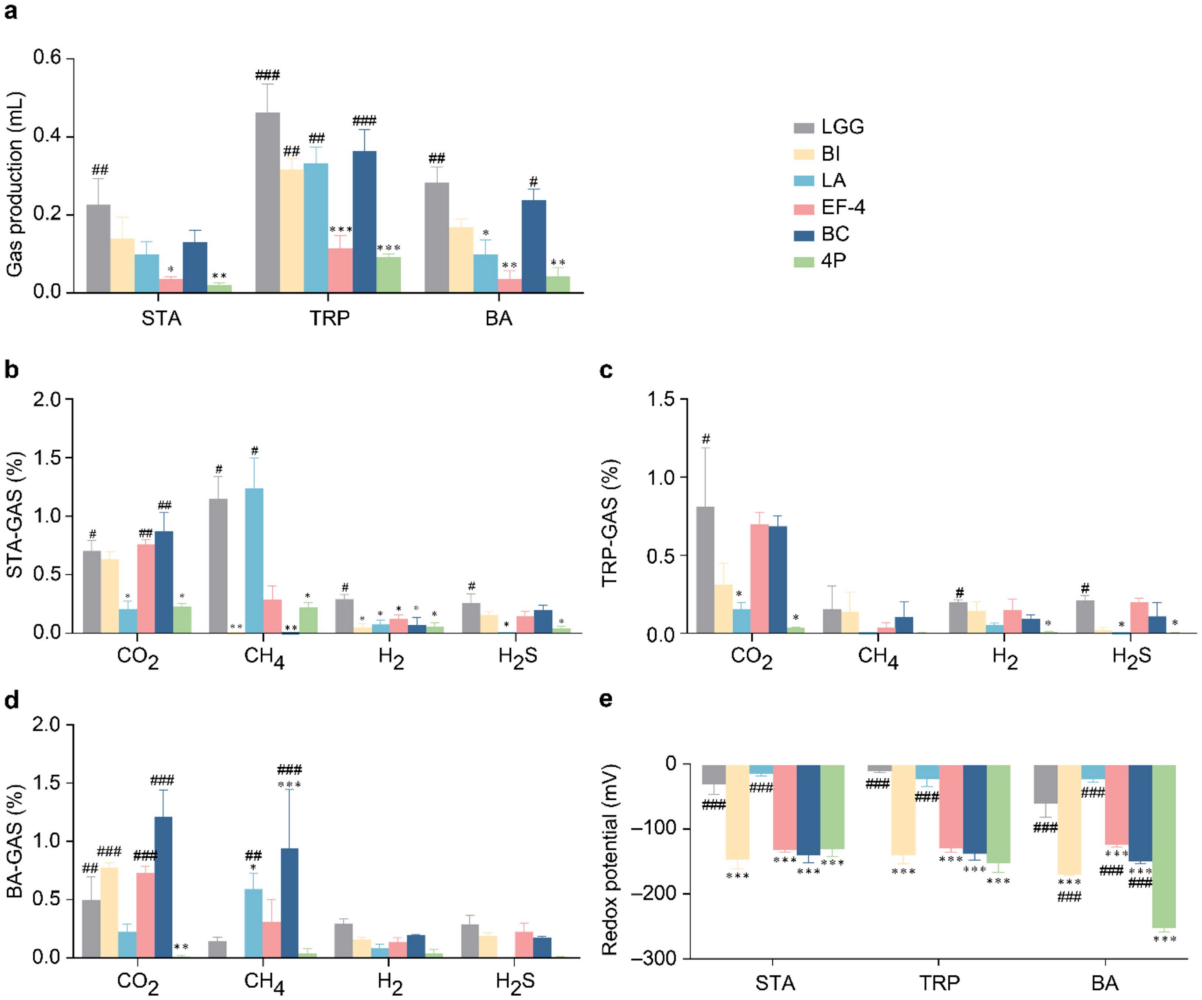
Gas metabolism and redox potential of different probiotics. **(a)** Gas production under STA, TRP, and BA culture conditions; **(b)** Gas metabolism under STA culture conditions; **(c)** Gas metabolism under TRP culture conditions; **(d)** Gas metabolism under BA culture conditions; **(e)** Redox potential under STA, TRP, and BA culture conditions. Data are presented as mean ± SEM, analyzed using a two-way ANOVA test. * vs. LGG, # vs. 4P; * *p* < 0.05, ** *p* < 0.01, *** *p* < 0.001; # *p* < 0.05, ## *p* < 0.01, ### *p* < 0.001.

Regarding gas production (Figure 4a), in STA medium, gas production in the 4P and EF-4 groups was significantly lower than that in the LGG group (*p* < 0.05). In TRP medium, gas production in the EF-4 group was significantly lower than that in the LGG group (*p* < 0.001), and the 4P group showed significantly lower gas production than the LGG, BI, LA, and BC groups (*p* < 0.01). In BA medium, gas production in the EF-4 and LA groups was significantly lower than that in the LGG group (*p* < 0.05), whereas gas production in the 4P group was significantly lower than that in the LGG and BC groups (*p* < 0.05). Regarding CO_2_ production in STA medium (Figure 4b), the LA group produced significantly lower CO_2_ levels than the LGG group (*p* < 0.05), whereas the BC, EF-4, and LGG groups produced significantly higher CO_2_ levels than the 4P group (*p* < 0.05). CH_4_ levels in the BI and BC groups were significantly lower than that in the LGG group (*p* < 0.05), and the 4P group had significantly lower CH_4_ levels than those in the LGG and LA groups (*p* < 0.05). H_2_ levels in the LGG group were significantly higher than those in the other groups (*p* < 0.05). H_2_S levels in the LA and 4P groups were significantly lower than that in the LGG group (*p* < 0.05). In TRP medium (Figure 4c), the CO_2_ levels in the LA and 4P groups were significantly lower than that in the LGG group (*p* < 0.05). The H_2_ and H_2_S levels in the 4P group were significantly lower than that in the LGG group (*p* < 0.05), and the H_2_S levels in the LA group were significantly lower than that in the LGG group (*p* < 0.05). In BA medium (Figure 4d), the CO_2_ levels in the 4P group were significantly lower than those in the LGG, BI, EF-4, and BC groups (*p* < 0.01). CH_4_ levels in the 4P group were significantly lower than those in the LA and BC groups (*p* < 0.05).

Regarding the redox potential (Figure 4e), in STA and TRP media, the redox potentials of the BI, BC, and EF-4 groups were significantly lower than that of the LGG group (*p* < 0.001). The redox potential in the 4P group was significantly lower than those in the LGG and BI groups (*p* < 0.001). In BA medium, the redox potentials of the BI, BC, and EF-4 groups were significantly lower than that of the LGG group (*p* < 0.001), and the redox potential of the 4P group was significantly lower than those of all the other groups (*p* < 0.001).

### 2.5 Establishing a colonic organoid culture model

**Figure 5:**
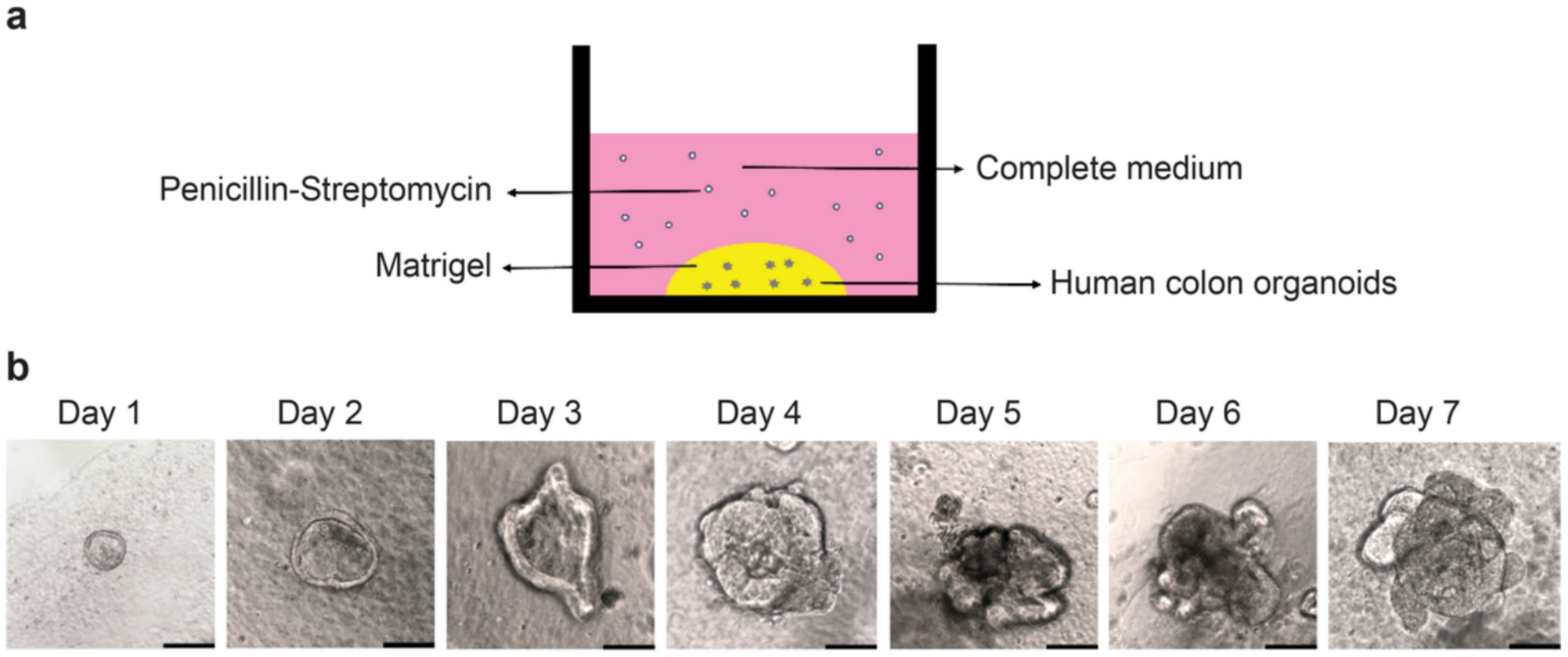
Construction of the human colonic organoid culture model. **(a)** Human colonic organoid culture model; **(b)** Observation of organoid growth from Day 1 to Day 7 under an optical microscope. Scale bar, 50 μm.

In the present study, we established a colonic organoid culture model (Figure 5a). Intestinal organoids were isolated from human colonic tissues and cultured in Matrigel. Organoids were seeded in domes in 24-well plates and began to bud on the third day of culture (Figure 5b).

### 2.6 Adhesive effect of probiotics on organoids

Based on the results of previous *in vitro* strain culture experiments, specific strains of the composite probiotic 4P were selected for subsequent organoid experiments. In probiotic adhesion study, organoids were cultured in traditional 3D Matrigel.

**Figure 6:**
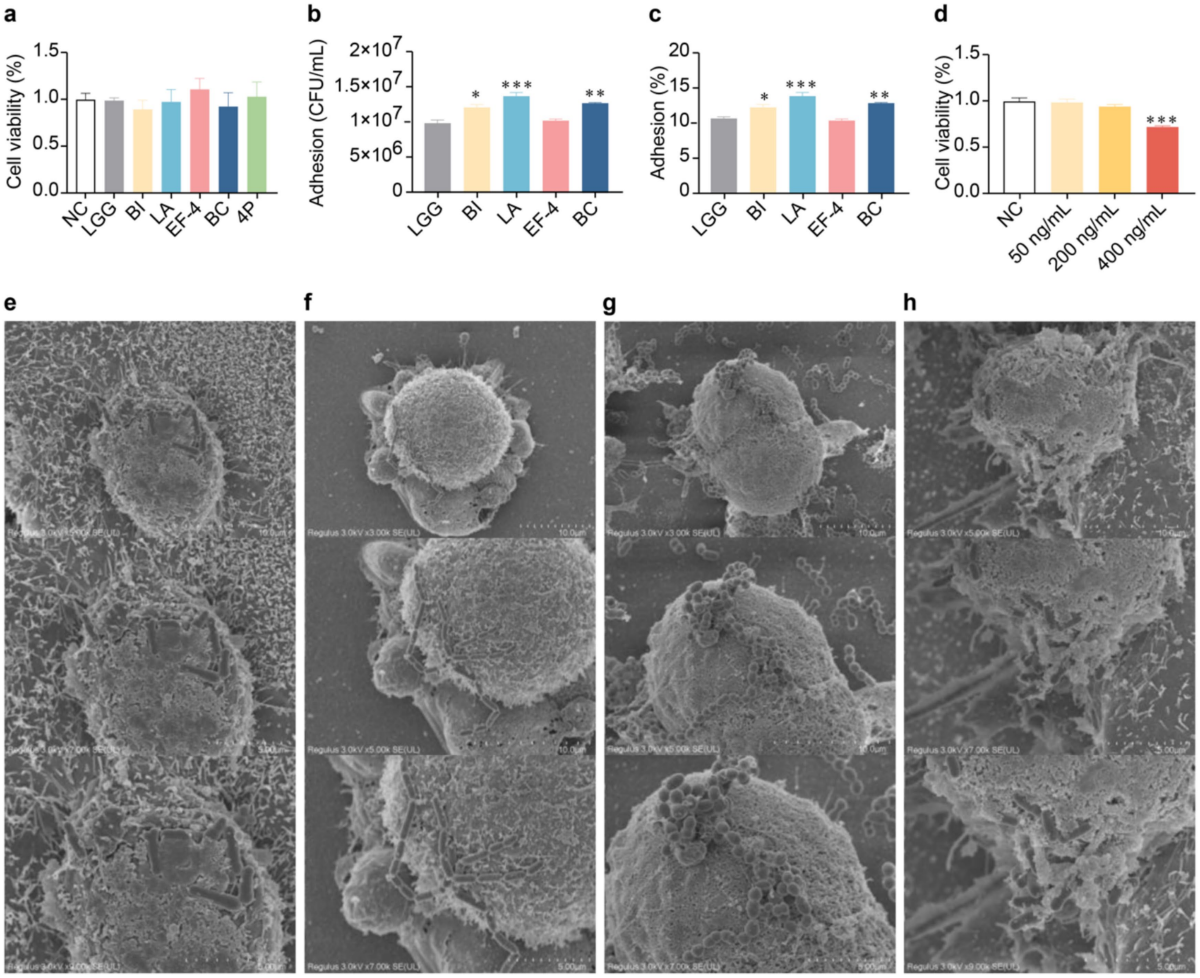
Adhesion of probiotics to colonic organoids. **(a)** Influence of different bacterial strains on organoid viability in conditioned media; **(b)** Adhesion of different bacterial strains to organoids; **(c)** Adhesion rate of different bacterial strains to organoids; **(d)** Influence of different concentrations of IFN-*γ* on organoid viability; **(e)** SEM image of infant *Bifidobacterium infantis* adhesion to organoids at a scale of 10 μm; **(f)** SEM image of *Lactobacillus acidophilus* adhesion to organoids at a scale of 5 μm; **(g)** SEM image of *Enterococcus faecium*-4 adhesion to organoids at a scale of 5 μm; **(h)** SEM image of *Bacucilius cereus* adhesion to organoids at a scale of 10 μm. Data are represented as mean ± SEM, analyzed using one-way ANOVA. **(b,c)** * vs. LGG, **(d)** * vs. NC; * *p* < 0.05, ** *p* < 0.01, *** *p* < 0.001.

The culture conditions of the various bacterial solutions showed no significant differences in organoid activity compared to the control group (Figure 6a). The adhesion levels of the BI, LA, and BC strains (Figures 6b,c) were significantly higher than that of the LGG strain (*p* < 0.05). Addition of interferon gamma (IFN-*γ*) at 400 ng/mL significantly reduced organoid activity (*p* < 0.001) (Figure 6d). Scanning electron microscopy (SEM) analysis was conducted on the individual strains within the 4P combination with good adhesion rates, including BI (Figure 6e), LA (Figure 6f), EF-4 (Figure 6g), and BC (Figure 6h). The SEM images show that all four probiotic strains exhibited good adhesion.

### 2.7 Effect of probiotics on inflammatory cytokines in organoids

Following IFN-γ treatment, the relative mRNA expression levels of ZO-1, E-cadherin, IL-1β, and IL-10 were assessed using real-time quantitative polymerase chain reaction (RT-qPCR). The results demonstrated that 200 ng/mL IFN-γ resulted in a significant decrease in ZO-1, E-cadherin, and IL-10 levels, while significantly increasing the level of IL-1β. These findings suggested that this concentration was suitable for subsequent experiments (Supplementary Figure S1).

**Figure 7:**
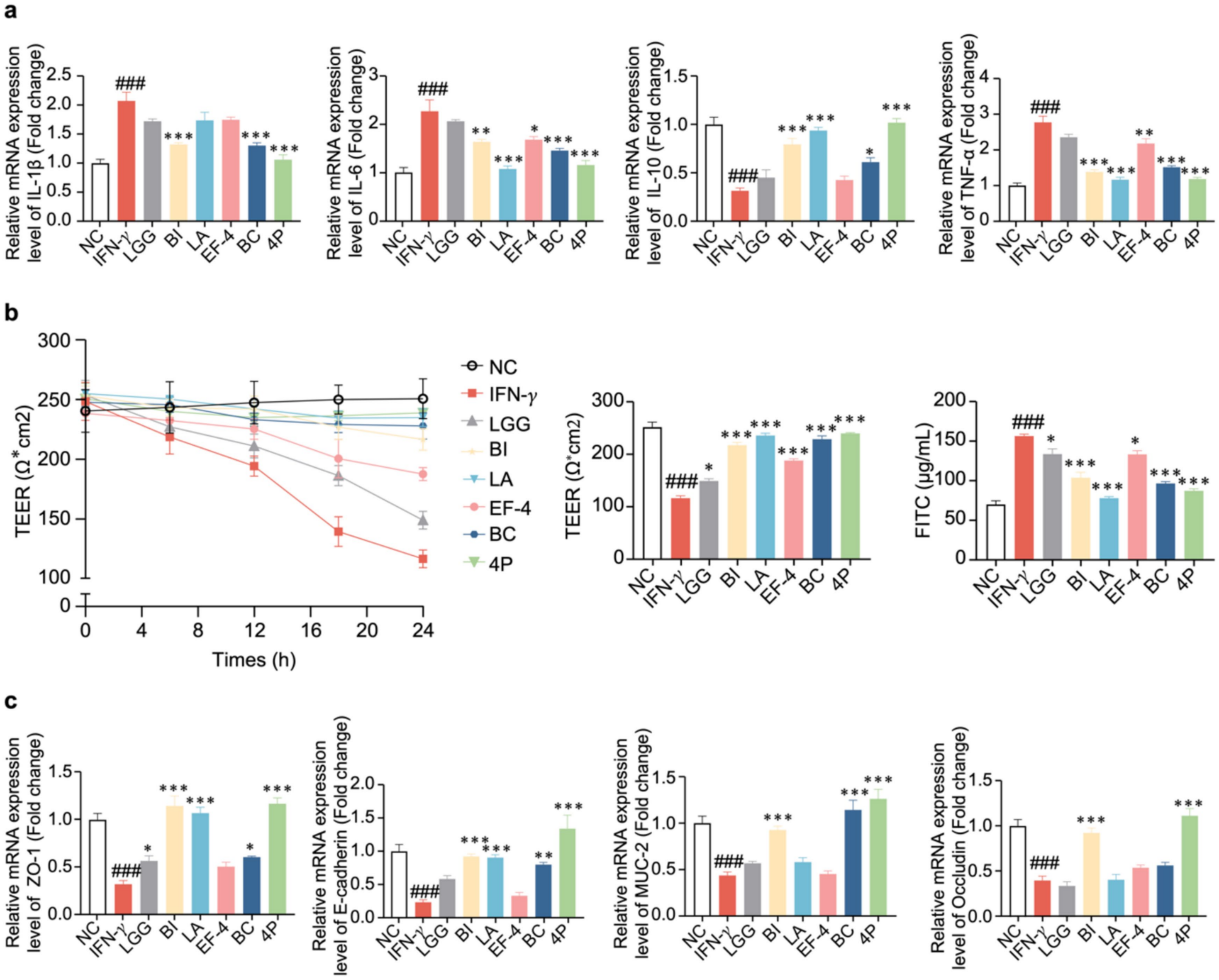
The effect of probiotics on cellular inflammatory cytokines and intestinal barrier. **(a)** IL-1β, IL-6, IL-10, TNF-*α*; **(b)** TEER in different time intervals of organoids and the concentration of FITC-dextran absorbed by the organoids. Data presented as means ± SEM, analyzed using one-way ANOVA. * *p* < 0.05, ** *p* < 0.01. *** *p* < 0.001; compared to the IFN-γ group, # *p* < 0.05, ## *p* < 0.01, ### *p* < 0.001. **(c)** The effect of probiotics on tight junction mRNA expression.

The relative mRNA expression levels of inflammatory cytokines in the organoids treated with the different probiotics were analyzed (Figure 7a). Compared to the control group, IFN-γ treatment significantly increased IL-1β, IL-6, and TNF-*α* levels (*p* < 0.001), while significantly decreasing the level of IL-10 (*p* < 0.001). Compared to the IFN-*γ* group, BI treatment significantly decreased the levels of IL-1β, IL-6, and TNF-α (*p* < 0.01), while significantly increasing the level of IL-10 (*p* < 0.05). LA treatment significantly decreased the levels of IL-6 and TNF-α (*p* < 0.001), while significantly increasing the level of IL-10 (*p* < 0.001). EF-4 treatment significantly decreased IL-6 and TNF-α levels (*p* < 0.05). BC treatment significantly decreased the level of IL-1β (*p* < 0.05), while significantly increasing the level of IL-10 (*p* < 0.05). 4P treatment significantly decreased IL-1β, IL-6, and TNF-α levels (*p* < 0.001), while significantly increasing the level of IL-10 (*p* < 0.001). There were no significant differences in the expression levels of IL-1β between the groups treated with BI, BC, and 4P and the control group. Similarly, there were no significant differences in the expression levels of IL-6 between the groups treated with LA, BC, and 4P and the control group. Furthermore, there were no significant differences in the expression levels of IL-10 between the groups treated with BI, LA, and 4P and the control group. Similarly, there were no significant differences in the expression levels of TNF-α between the groups treated with BI, LA, and 4P and the control group.

### 2.8 Impact of probiotics on organoid permeability

To evaluate the role of probiotics in intestinal mucosal barrier function, we quantified fluorescein isothiocyanate (FITC)-dextran permeability and transendothelial electron resistance (TEER) in organoids with IFN-*γ*-induced mucosal damage. The TEER values for each group at various timepoints from 0 to 24 h are shown in Figure 7b. After 24 h, IFN-γ significantly decreased TEER compared to that in the control group (*p* < 0.001), whereas the LGG, BI, LA, EF-4, BC, and 4P groups demonstrated a significant reversal in this decline (*p* < 0.05). No significant differences were observed between the LA, BC, and 4P groups and the control group. Additionally, IFN-γ significantly increased FITC levels compared to that in the control group (*p* < 0.001), whereas the LGG, BI, LA, EF-4, BC, and 4P groups demonstrated a significant reversal in this change (*p* < 0.05). The LA and 4P groups showed no significant differences in FITC levels compared to the control group.

### 2.9 Impact of probiotics on tight junction proteins in organoids

Using RT-qPCR, the relative mRNA expression levels of tight junction proteins in the organoids in response to the different probiotics were examined (Figure 7c). Compared to the control group, IFN-*γ* treatment significantly decreased ZO-1, ECAD, MUC-2, and OCLN levels (*p* < 0.001). Compared to the IFN-γ treatment, BI treatment significantly increased ZO-1(by 256%), E-cadherin (by 288%), MUC-2 (by 112%), and Occludin (by 129%) levels (*p* < 0.001). LA treatment significantly increased (*p* < 0.01) ZO-1 and E-cadherin levels, whereas BC treatment significantly increased ZO-1, E-cadherin, and MUC-2 levels (*p* < 0.01). After treatment with 4P, there was a significant increase in the levels of ZO-1, E-cadherin, MUC-2, and occludin (*p* < 0.001). There were no significant differences in the expression levels of ZO-1 after treatment with BI, LA, or 4P compared to those in the control group. Similarly, there were no significant differences in the expression levels of E-cadherin after treatment with LGG, BI, LA, BC, or 4P compared to those in the control group. Additionally, there were no significant differences in the expression levels of MUC-2 after treatment with BI, BC, or 4P compared to those in the control group. There were also no significant differences in the expression levels of occludin after treatment with BI and 4P compared with those in the control group.

**Figure 8:**
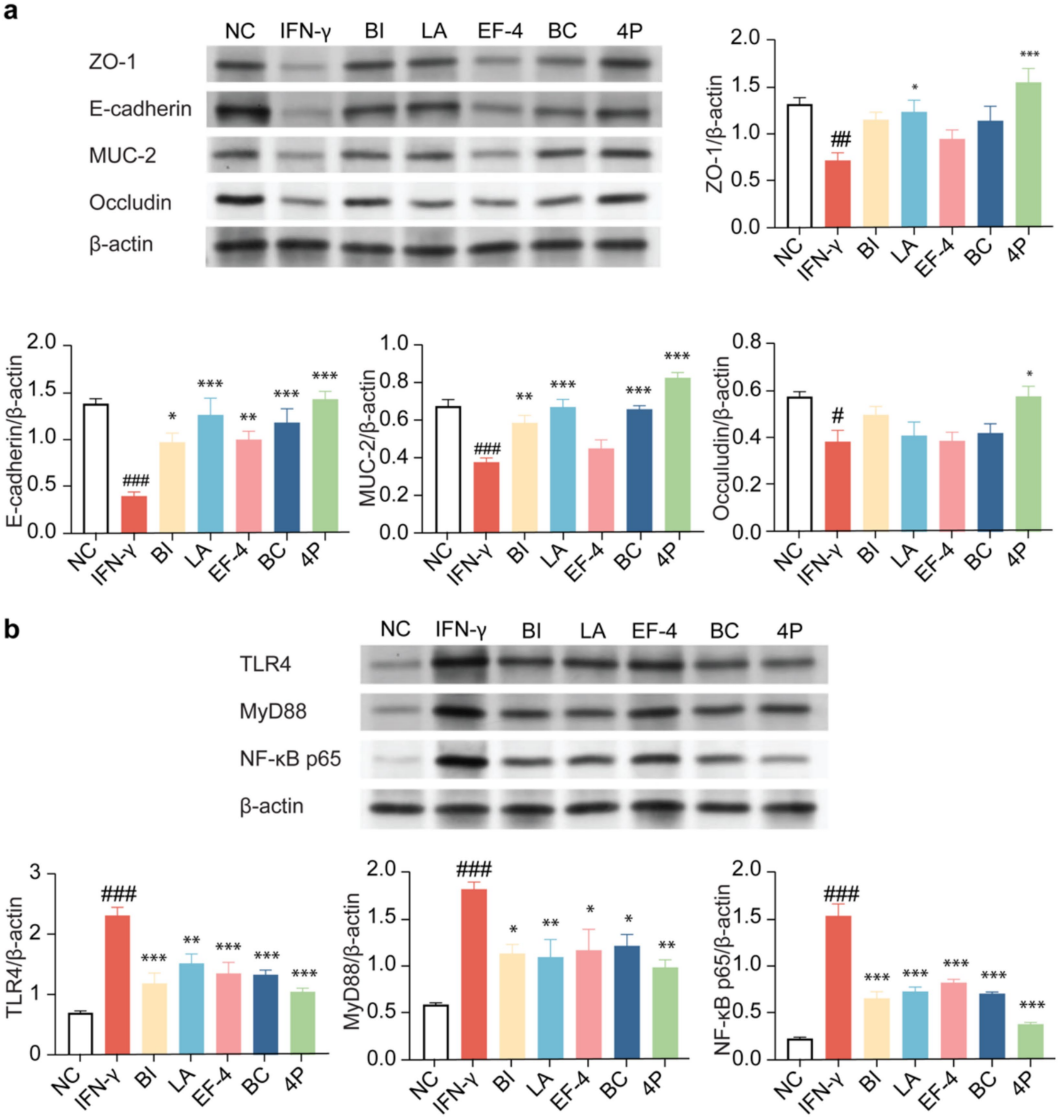
The impact of different probiotics on the tight junction proteins and NF-κB pathway in organoids. **(a)** Expression of tight junction proteins. **(b)** Influence on the NF-κB pathway. Compared to the NC group * *p* < 0.05, ** *p* < 0.01. *** *p* < 0.001; Compared to the IFN-γ group, # *p* < 0.05, ## *p* < 0.01, ### *p* < 0.001.

The expression levels of tight junction proteins in the organoids were determined using western blotting (Figure 8a). Compared to the control group, IFN-γ treatment significantly decreased the expression levels of tight junction proteins ZO-1, E-cadherin, MUC-2, and occludin (*p* < 0.001), whereas BI treatment significantly increased the expression levels of E-cadherin and MUC-2 (*p* < 0.05). LA significantly increased the expression levels of ZO-1, E-cadherin, and MUC-2 (*p* < 0.05). EF-4 significantly increased the expression levels of E-cadherin (*p* < 0.01), whereas BC significantly increased the expression levels of E-cadherin and MUC-2 (*p* < 0.001). For 4P, the intervention effect was the most significant, significantly increasing the expression levels of ZO-1, E-cadherin, MUC-2, and occludin (*p* < 0.05). Meanwhile, BI, LA, and BC showed significantly higher expression levels of TLR4, MyD88, and NF-κB p65 (*p* < 0.05) than the model group, and the intervention effect of 4P was significantly better than that of other groups (*p* < 0.001) (Figure 8b).

## 3 Discussion

IBD is a multifactorial disorder, for which no effective treatment is currently available. Therefore, the identification of new therapeutic strategies to reduce the incidence of IBD is crucial. One significant characteristic of IBD is a compromised intestinal barrier, accompanied by heightened production of inflammatory cytokines and impaired tight junction integrity (Dolinger et al., 2024). In previous studies, probiotic supplements, particularly composite probiotics containing strains of the *Bifidobacterium* and *Lactobacillus* genera, were clinically proven effective in patients with ulcerative colitis (Bai et al., 2023; Saez-Lara et al., 2015). However, the specific mechanisms of action remain unclear. Therefore, additional investigation is necessary to further understand how composite probiotics affect the intestinal barrier and immune function.

Anaerobic microorganisms in the host intestine produce large amounts of SCFAs via fermentation, consisting of acetate, propionate, and butyrate. SCFAs are absorbed by colonic cells and influence intestinal motility, intestinal barrier function, and host metabolic regulation. Concurrently, they also participate in immune regulation, cell apoptosis, and inflammatory responses (Wu et al., 2024; Tao and Wang, 2024). Acetate can serve as a source of energy for the gut microbiota and promote the development of a favourable acidic environment in the intestine, which supports the growth and multiplication of beneficial bacteria. Propionates lower serum cholesterol levels and reduce inflammation. In previous studies, anaerobic bacteria (such as *Clostridium* and *Bacteroides*) were shown to effectively produce SCFAs, especially butyrate (Parada Venegas et al., 2019), whereas lactate and acetate salts produced by *Bifidobacterium* may serve as substrates for colonic bacteria to produce butyrate (Moens et al., 2017). A decrease in certain anaerobic bacteria and the increased susceptibility of the intestines are closely related to decreased butyrate, leading to an increase in the intestinal oxygen content and aerobic bacteria (such as *Escherichia coli*) (Rivera-Chavez et al., 2016).

In this study, the production levels of acetate, propionate, and butyrate were significantly higher in the four bacterial strains than those in the standard LGG strain. The production of acetate, propionate, and butyrate by the composite probiotic 4P was significantly higher than that of 3P, 2P, and 1P, and also higher than that of the LGG standard strain. Compared to single probiotic interventions, the composite probiotic 4P exhibited a higher production of SCFAs, possibly because of the specific interactions among the four probiotic strains in this study, such as bacterial cross-feeding relationships, achieving a synergistic effect.

An elevation in gas production is directly linked to a disruption in gut microbiota. Once gas-producing bacteria lose their balance, excess gas is produced, leading to increased intestinal pressure. This not only increases the risk of intestinal diseases such as gastrointestinal dysfunction and inflammation but also leads to abdominal pain, bloating, and diarrhea (Kalantar-Zadeh et al., 2019). Although most intestinal gases are absorbed into the bloodstream and expelled from the body through pulmonary circulation, they still affect the physiological health of the body. Excessive gases may affect the expansion of the colonic intestinal wall, thereby affecting the transport of substances (Azpiroz, 2005). Methane production may also slow gastrointestinal transit and decrease serotonin levels, leading to irritable bowel syndrome or constipation (Seo et al., 2013). In this study, the composite probiotic 4P significantly reduced gas production, which was reflected in the generation of harmful gases such as CO_2_, CH_4_, and H_2_S.

The oxidation–reduction potential is a crucial indicator of the complex environment in the intestine and significantly influences host health. This macroscopically reflects the reaction between the intestinal microbiota and intestinal health. Colonization by anaerobic probiotics is challenging in both inflammatory and aerobic environments. When intestinal dysbiosis occurs, some electron acceptors, such as O_2_ and NO_2_, prefer metabolic reactions with higher oxidation–reduction potentials based on thermodynamic laws, thereby reflecting changes in intestinal inflammation and microbial community structure (Penumutchu et al., 2023). In this study, the probiotic combination of 4P demonstrated the best ability to regulate oxidation–reduction potential. This may be due to the initial colonization of facultative anaerobic bacteria with a high oxidation–reduction potential in the intestine. As oxygen becomes less available with the proliferation of facultative anaerobic bacteria, strictly anaerobic bacteria emerge. When a low-oxygen environment is formed, which is associated with a decrease in the oxidation–reduction potential, the passive transport of intestinal epithelial cells promotes an increase in oxygen content, facilitating the growth of aerobic bacteria. Therefore, intestinal tight junctions may play an important role in this process.

Adherence to the intestinal mucosal layer is crucial for probiotics to exert their physiological effects. In this study, LA, BC, and BI exhibited significantly higher adhesion than the standard LGG strain. Adhesive proteins on the surface of probiotics, such as S-layer proteins, slave sortase proteins, and anchor-free motif proteins, specifically adhere to the intestinal epithelial cells. The probiotics in the 4P combination demonstrated excellent self-aggregation ability under SEM, which is believed to have certain benefits in eliminating pathogens (Mazhar et al., 2023).

The intestinal barrier selectively absorbs various nutrients, while preventing harmful microbes and pathogens from entering the host. Intestinal epithelial tissue consists of a single layer of cells that are connected by tight junctions, specifically Occludin, E-cadherin, and ZO-1. When the integrity of the intestinal barrier is weakened, leading to “leaky gut,” it can trigger local inflammation in the intestine and further pathological reactions. Hence, it is crucial to enhance food health and nutrition by preserving the integrity of the intestinal barrier through the use of probiotics in the diet. Within this investigation, a co-culture model of probiotics and gut organoids was employed. The ability of the composite probiotic 4P and its four individual strains to maintain TEER was compared. Compared to the control group, 4P treatment significantly reduced the decrease in TEER induced by IFN-*γ*. Additionally, the individual strains exhibited excellent ability to mitigate the decrease in TEER. In the study of intestinal permeability using FITC-dextran. Furthermore, it was noted that the composite probiotic 4P effectively decreased the rise in intestinal permeability caused by IFN-γ, consequently, the integrity of the intestinal barrier tissue is being restored. The composite probiotic 4P demonstrates exceptional capability in protecting the intestinal barrier, consequently maintaining the integrity of the intestinal mucosa.

Intestinal barrier damage often accompanies inflammation, oxidative stress, and other phenomena (Cao et al., 2020). The study revealed that the composite probiotic 4P had a substantial impact on the levels of IL-1β, IL-6, TNF-*α*, and IL-10, resulting in a reduction of the former three and an increase of the latter. Previous research has indicated a crucial role for the interaction between inflammasomes and the intestinal microbiota in maintaining intestinal homeostasis and regulating immune responses (Zmora et al., 2017). Modulation of downstream inflammasome mediators (like IL-1β and IL-8) may be a promising intervention for gastrointestinal diseases. IL-10 is a cytokine with anti-inflammatory properties that has a significant effect in repairing the tight junctions in the intestines. A further study indicated that the makeup of the gut microbiota in mice lacking the IL-10 protein was notably different from that of the control group. and the IL-10 deficient mice spontaneously developed symptoms of colitis over time, which were reversed upon IL-10 supplementation (Zegarra Ruiz et al., 2022). This finding highlights the important role of IL-10 in suppressing intestinal inflammation. The composite probiotic 4P exhibited excellent intestinal barrier protection based on the analysis of tight junction mRNA levels.

ZO-1 plays a crucial role in controlling the establishment of tight junctions and acts as a framework connecting actin and the cytoskeleton (Kuo et al., 2022). E-cadherin and occludin are key tight junction proteins, and their interactions maintain tight junction integrity. MUC-2 is the most abundant mucin in the small intestine (Zeisel et al., 2019). Immunoblotting analysis also revealed that the composite probiotic 4P substantially enhanced the expression of tight junction proteins. Studies have indicated that composite probiotics improve the function of the intestinal barrier by enhancing the production of tight junction proteins and mucins (Wang et al., 2020; Slifer and Blikslager, 2020). Consequently, it is crucial to enhance the production of tight junction proteins and mucins in order to restore the function of the intestinal barrier.

To further explore the mechanism of action, we investigated relevant proteins and upstream and downstream factors in the NF-κB pathway. Previous investigation has shown that TLR4, a regulatory factor in the NF-κB pathway, plays a crucial role in intestinal barrier damage and inflammation (Zhou et al., 2018). In this investigation, the protein expression levels of TLR4 and NF-κB were dramatically lowered by the composite probiotic 4P, when compared to the model group., indicating that it significantly reduced the inflammatory response. Additionally, evidence suggests that SCFAs (such as propionic and butyric acids) produced by probiotics have inhibitory effects on the NF-κB pathway and can effectively ameliorate intestinal inflammation (Zhang et al., 2023; Sun et al., 2021). This finding suggests that the excellent SCFA production capability of the composite probiotic 4P may have contributed to the observed results. TLR4 activates downstream inflammatory signals through MyD88 nodes, therefore facilitating the release of inflammatory cytokines and triggering the NF-κB pathway. In this investigation, the protein expression levels of MyD88 significantly decreased after intervention with the composite probiotic 4P compared to the model group, further indicating that the protective effect of the composite probiotic 4P on the intestinal barrier may be achieved through the NF-κB pathway.

At the same time, there are still some limitations that cannot be ignored in this study, such as the room for improvement in the sample size of the experiment. Further improvement is needed for the above issues in the future.

In conclusion, this investigation provides insight into the possible therapeutic use of composite probiotics in reducing the malfunctioning of the intestinal barrier that is linked to several gastrointestinal illnesses. Through a series of *in vitro* experiments using a co-culture model of probiotics and intestinal organoids, we demonstrated that the composite probiotic 4P effectively enhanced intestinal barrier integrity, reduced inflammation, and regulated the NF-κB signaling pathway. Our findings suggest the potential of probiotic supplementation in maintaining gut health and preserving intestinal barrier integrity. The discovered impacts of 4P on enhancing the production of tight junction proteins, decreasing pro-inflammatory cytokines, and regulating NF-κB pathway activity offer vital understanding into its mode of operation. Overall, this study adds to the increasing amount of evidence that supports the therapeutic benefits of composite probiotics for treating gastrointestinal diseases that are characterised by disruption of the intestinal barrier. Additional investigation is necessary to examine the practical consequences and lasting impacts of probiotic treatments in human subjects.

## 4 Materials and methods

### 4.1 Probiotics, probiotic combinations, and culture media

The probiotic strains selected for this study were the commercial probiotic LGG (ATCC 53103); 1P, including *Bacillus licheniformis*; 2P, including *Enterococcus faecium* and *B. subtilis*; 3P, including *Bifidobacterium longum*-3, *E. faecalis*-3, and *Lactobacillus plantarum*; and 4P, including BI (GMCC 0460.1), LA (GMCC 0460.2), EF-4 (GMCC 0460.3), and BC (GMCC 0460.4). All the probiotic strains were obtained from China General Microbiological Culture Collection Center (GMCC).

After preparing YCFA (yeast extract, casitone, fatty acids) culture medium, 8 g/L starch, 5 mMol/L *L*-tryptophan, and 5 μL/mL bile acids were separately added to create STA, TRP, and BA media, respectively.

### 4.2 Anaerobic fermentation of the strains

The probiotics were inoculated into liquid culture medium for activation. The growth status was monitored by measuring the optical density until the cells reached logarithmic growth phase. Once this phase was reached, the bacterial suspension was adjusted to 10^8^ colony-forming units/mL using a McFarland turbidity tube and set aside for further use. Next, 500 μL of the probiotic suspension was added to 5 mL of each of the three culture media types, with three replicates for each. Following anaerobic fermentation, subsequent analyses were conducted at a temperature of 37°C for a duration of 24 h in a temperature-controlled incubator.

### 4.3 SCFA analysis

The SCFAs produced by probiotic strains after anaerobic fermentation, including acetic, propionic, butyric, isobutyric, valeric, and isovaleric acids, were detected using a GC9720II gas chromatograph (Fuli, Inc., Zhejiang, China). The gas chromatograph was equipped with a flame ionisation detector and an Agilent-FFAP column, which had operating temperature range of 75–220°C and dimensions of 30 m × 0.25 mm × 0.25 μm (Calvigioni et al., 2023).

### 4.4 Gas production and gas type analysis

The gas composition and content produced after fermentation were determined using an HL-QT01 intestinal microbiota fermentation gas analyzer (Hailu Biotechnology Co., Ltd., Suzhou, Jiangsu Province, China) (Rose et al., 2021).

### 4.5 Oxidation–reduction potential

The oxidation–reduction potential of the samples after fermentation was measured using an Eutech™ pH 700 meter (Thermo Fisher Scientific, Waltham, MA, United States) (Penumutchu et al., 2023).

### 4.6 Colonic organoid culture

The human colonic organoids utilised in this investigation were supplied by the First Affiliated Hospital of Zhejiang University (Deidentified tissues). The cultivation of organoids is based on previous literature (Hou et al., 2018)^,^ in short, the colonic organoids were cultured on Matrigel (Corning, NY, United States) in 24-well plates, with 50 μL of Matrigel allocated to each well. The plates were then incubated at 37°C for 20 min to allow the Matrigel to solidify. Subsequently, 750 μL of colonic organoid growth medium (06010, STEMCELL, Shanghai, China) was added to each well. The growth medium was changed thrice weekly, and passaging was conducted 7–14 days after sowing. For 2D cultivation of organoids, they are first cultured in a 3D matrix (such as Matrigel) until they reach the appropriate size. Then use a mild cell dissociation reagent to blow apart the Matrigel dome structure. Collect 2–3 domes (containing approximately 100–150 organoids) and inoculate them into IntestiCultTM monolayer growth medium (24 well transwell culture plate). For barrier function measurement, TEER and FITC permeability measurements are performed after the formation of a stable monolayer of cells.

The organoids were pretreated with 200 ng/mL of IFN-*γ* (Merck, Germany) for 12 h to establish the inflammation model, with this concentration validated through preliminary experiments (Supplementary Figure S1). Subsequently, different probiotics (1 × 10^6 CFU/well) were co-cultured with the organoids for 24 h (MOI = 10,000:1). The control group was treated with complete medium instead of probiotics. After co-cultivation, samples were collected for subsequent experimental analysis.

### 4.7 SEM analysis

For the adhesion assay, probiotics were microinjected into the organoids. Briefly, organoids were cultured in Matrigel until they reached the desired stage. Probiotic suspensions (10^7 CFU/mL) were prepared. Using a microinjection setup, a small volume of probiotic suspension was injected directly into the apical surface of the organoids under a confocal microscope. After injection, organoids were incubated at 37°C with 5% CO2 for 24h. Probiotic adhesion was assessed using bacterial counting (Li et al., 2019).

The morphology of probiotic adhesion to the organoids was observed using a field-emission SEM (Regulus 8,100, Hitachi High-tech Corporation of Tokyo, Japan). The samples were adhered to a conductive adhesive and coated with a layer of gold under vacuum to prepare micrographs.

### 4.8 RT-qPCR analysis

Following the respective treatments, colonic organoids were collected and total RNA was extracted using a TransZol Up Plus RNA Kit (Elbadawi et al., 2021) (ER501, TransGen Biotech, Hangzhou, China). Subsequently, gDNA was removed and the TransScript^®^ One-Step gDNA Removal and cDNA Synthesis SuperMix Kit was utilised for cDNA synthesis (AT314, TransGen Biotech, Hangzhou, China). The RT-qPCR analysis was conducted using the PerfectStart^®^ Green qPCR SuperMix (AQ601, TransGen Biotech, Hangzhou, China) as per the manufacturer’s guidelines. The Bio-Rad sequence detection system was used to monitor the process. The 2−∆∆Ct technique was used to calculate the relative transcript levels. The experiment was replicated a minimum of three times. The primer sequences utilised for RT-qPCR are enumerated in Table 1.

**Table 1:** Primer sequences used for RT-qPCR.

Primer ID	Sequences (5′-3′)
IL-1β-F (Human)	TAAGCCCACTCTACAGCTGG
IL-1β-R (Human)	GAGAGGTGCTGATGTACCAG
IL-6-F (Human)	CAATGAGGAGACTTGCCTGG
IL-6-R (Human)	GCACAGCTCTGGCTTGTTCC
IL-10-F (Human)	CATAAATTAGAGGTCCAAAATCG
IL-10-R (Human)	AAGGGGCTGGGTCAGCTAT
TNF-α-F (Human)	TTCCAGAAGATGATCTGATGC
TNF-α-R (Human)	TCAGCCTCTTCTCCTTCCT
ZO-1-F (Human)	GTTGGTACGGTGCCCTGAAAGA
ZO-1-R (Human)	GCTGACAGGTAGGACAGACGAT
E-cadherin-F (Human)	GCACATATGTAGCTCTCATC
E-cadherin-R (Human)	CCTTCACAGTCACACACATG
MUC2-F (Human)	CTGCACCAAGACCGTCCTCATG
MUC2-R (Human)	GCAAGGACTGAACAAAGACTCAGA
Occludin-F (Human)	TGCATGTTCGACCAATGC
Occludin-R (Human)	AAGCCACTTCCTCCATAAGG
GAPDH-F (Human)	ATGACCTTGCCACAGCC
GADPH-R (Human)	CCCATCACCATCTTCCAG

### 4.9 Western blotting analysis

Colonic organoids were placed in 6-well plates with a cell density of 1 × 10^6^ cells per well. The cellular protein content was quantified by performing protein extraction using a BCA Protein Assay Kit (P0012, Beyotime, Shanghai, China). Afterwards, the proteins were isolated by employing 8–15% sodium dodecyl sulfate-polyacrylamide gel electrophoresis and subsequently deposited onto polyvinylidene difluoride membranes (LC2007, Thermo Fisher, Waltham, MA, United States) at a voltage of 80 V for a duration of 1.5 h. The membranes were washed twice with Milli-Q water, then blocked with skim milk. Next, the membranes were incubated overnight at 4°C with the following primary antibodies: ZO-1 (1:1000), E-cadherin (1:1000), MUC-2 (1:500), occludin (1:500), TLR4 (1:1000), MyD88 (1:1000), and NF-κB p65 (1:500). Afterwards, the membranes underwent four washes with Tris-buffered saline containing 0.1% Tween 20. Subsequently, the membranes were incubated with goat anti-mouse IgG (H + L) (1:5000, A0216, Beyotime) or goat anti-rabbit IgG (H + L) (1:5000, A0208, Beyotime) at a temperature of 37°C for a duration of 45 min. The bands were seen after undergoing six rounds of washing (Cao et al., 2018).

### 4.10 Statistical analysis

The experimental data is shown either as the mean ± standard error of the mean or in the form of box-and-whisker plots. The statistical analyses were conducted using IBM SPSS Statistics 26 and GraphPad Prism software (version 9.0.0). The differences between groups were analyzed using one-way analysis of variance and Duncan’s test. The threshold for statistical significance was established at a *p*-value of less than 0.05. The following symbols are used: * for *p* < 0.05, ** for *p* < 0.01, *** for *p* < 0.001.

## Data Availability

The raw data supporting the conclusions of this article will be made available by the authors, without undue reservation.

## Supplementary material

SUPPLEMENTARY FIGURE S1 Effect of different concentrations of IFN-γ on the expression of inflammatory cytokines and tight junction mRNA. **(a)** ZO-1; **(b)** E-cadherin; **(c)** IL-β; **(d)** IL-10. Data presented as means ± SEM, analyzed using one-way ANOVA. * *p* < 0.05, ** *p* < 0.01, *** *p* < 0.001.

## References

[ref1] Azpiroz F. (2005). Intestinal gas dynamics: mechanisms and clinical relevance. Gut 54, 893–895. doi: 10.1136/gut.2004.048868, PMID: 15951528 PMC1774596

[ref2] Bai T. Xu Z. Xia P. Feng Y. Liu B. Liu H.. (2023). The short-term efficacy of Bifidobacterium quadruple viable tablet in patients with diarrhea-predominant irritable bowel syndrome: potentially mediated by metabolism rather than diversity regulation. Am. J. Gastroenterol. 118, 1256–1267. doi: 10.14309/ajg.0000000000002147, PMID: 36717369

[ref3] Calvigioni M. Bertolini A. Codini S. Mazzantini D. Panattoni A. Massimino M.. (2023). HPLC-MS-MS quantification of short-chain fatty acids actively secreted by probiotic strains. Front. Microbiol. 14:1124144. doi: 10.3389/fmicb.2023.1124144, PMID: 36937254 PMC10020375

[ref4] Cao H. Liu J. Shen P. Cai J. Han Y. Zhu K.. (2018). Protective effect of Naringin on DSS-induced ulcerative colitis in mice. J. Agric. Food Chem. 66, 13133–13140. doi: 10.1021/acs.jafc.8b03942, PMID: 30472831

[ref5] Cao S. Wang C. Yan J. Li X. Wen J. Hu C. (2020). Curcumin ameliorates oxidative stress-induced intestinal barrier injury and mitochondrial damage by promoting Parkin dependent mitophagy through AMPK-TFEB signal pathway. Free Radic. Biol. Med. 147, 8–22. doi: 10.1016/j.freeradbiomed.2019.12.004 31816386

[ref6] Chen Y. Feng S. Li Y. Zhang C. Chao G. Zhang S. (2024). Gut microbiota and intestinal immunity-a crosstalk in irritable bowel syndrome. Immunology 172, 1–20. doi: 10.1111/imm.13749, PMID: 38174581

[ref7] Dolinger M. Torres J. Vermeire S. (2024). Crohn's disease. Lancet 403, 1177–1191. doi: 10.1016/S0140-6736(23)02586-2, PMID: 38437854

[ref8] Elbadawi M. Ammar R. M. Aziz-Kalbhenn H. Rabini S. Klauck S. M. Dawood M.. (2021). Anti-inflammatory and tight junction protective activity of the herbal preparation STW 5-II on mouse intestinal organoids. Phytomedicine 88:153589. doi: 10.1016/j.phymed.2021.153589, PMID: 34111617

[ref9] Fan L. Qi Y. Qu S. Chen X. Li A. Hendi M.. (2021). B. adolescentis ameliorates chronic colitis by regulating Treg/Th2 response and gut microbiota remodeling. Gut Microbes 13, 1–17. doi: 10.1080/19490976.2020.1826746, PMID: 33557671 PMC7889144

[ref10] Giri R. Hoedt E. C. Khushi S. Salim A. A. Bergot A. S. Schreiber V.. (2022). Secreted NF-kappaB suppressive microbial metabolites modulate gut inflammation. Cell Rep. 39:110646. doi: 10.1016/j.celrep.2022.110646, PMID: 35417687

[ref11] Han H. You Y. Cha S. Kim T. R. Sohn M. Park J. (2023). Multi-species probiotic strain mixture enhances intestinal barrier function by regulating inflammation and tight junctions in lipopolysaccharides stimulated Caco-2 cells. Microorganisms 11:656. doi: 10.3390/microorganisms11030656, PMID: 36985228 PMC10056128

[ref12] He X. Ye G. Xu S. Chen X. He X. Gong Z. (2023). Effects of three different probiotics of Tibetan sheep origin and their complex probiotics on intestinal damage, immunity, and immune signaling pathways of mice infected with Clostridium perfringens type C. Front. Microbiol. 14:1177232. doi: 10.3389/fmicb.2023.1177232, PMID: 37138630 PMC10149710

[ref13] Hou Q. Ye L. Liu H. Huang L. Yang Q. Turner J. R.. (2018). Lactobacillus accelerates ISCs regeneration to protect the integrity of intestinal mucosa through activation of STAT3 signaling pathway induced by LPLs secretion of IL-22. Cell Death Differ. 25, 1657–1670. doi: 10.1038/s41418-018-0070-2, PMID: 29459771 PMC6143595

[ref14] Kalantar-Zadeh K. Berean K. J. Burgell R. E. Muir J. G. Gibson P. R. (2019). Intestinal gases: influence on gut disorders and the role of dietary manipulations. Nat. Rev. Gastroenterol. Hepatol. 16, 733–747. doi: 10.1038/s41575-019-0193-z, PMID: 31520080

[ref15] Kuo W. T. Odenwald M. A. Turner J. R. Zuo L. (2022). Tight junction proteins occludin and ZO-1 as regulators of epithelial proliferation and survival. Ann. N. Y. Acad. Sci. 1514, 21–33. doi: 10.1111/nyas.14798, PMID: 35580994 PMC9427709

[ref16] Li C. Bei T. Niu Z. Guo X. Wang M. Lu H.. (2019). Adhesion and colonization of the probiotic Lactobacillus rhamnosus labeled by Dsred2 in mouse gut. Curr. Microbiol. 76, 896–903. doi: 10.1007/s00284-019-01706-8, PMID: 31115599

[ref17] Liu P. Liu Z. Wang J. Wang J. Gao M. Zhang Y.. (2024). Immunoregulatory role of the gut microbiota in inflammatory depression. Nat. Commun. 15:3003. doi: 10.1038/s41467-024-47273-w, PMID: 38589368 PMC11001948

[ref18] Mazhar S. Simon A. Khokhlova E. Colom J. Leeuwendaal N. Deaton J.. (2023). In vitro safety and functional characterization of the novel Bacillus coagulans strain CGI314. Front. Microbiol. 14:1302480. doi: 10.3389/fmicb.2023.1302480 38274758 PMC10809412

[ref19] Moens F. Verce M. De Vuyst L. (2017). Lactate- and acetate-based cross-feeding interactions between selected strains of lactobacilli, bifidobacteria and colon bacteria in the presence of inulin-type fructans. Int. J. Food Microbiol. 241, 225–236. doi: 10.1016/j.ijfoodmicro.2016.10.019 27810444

[ref20] Mukherjee T. Kumar N. Chawla M. Philpott D. J. Basak S. (2024). The NF-kappaB signaling system in the immunopathogenesis of inflammatory bowel disease. Sci. Signal. 17:eadh1641. doi: 10.1126/scisignal.adh1641, PMID: 38194476

[ref21] Murali S. K. Mansell T. J. (2024). Next generation probiotics: engineering live biotherapeutics. Biotechnol. Adv. 72:108336. doi: 10.1016/j.biotechadv.2024.108336, PMID: 38432422

[ref22] Naveed U. Jiang C. Yan Q. Wu Y. Zhao J. Zhang B.. (2024). Inhibitory effect of Lactococcus and Enterococcus faecalis on Citrobacter colitis in mice. Microorganisms 12:730. doi: 10.3390/microorganisms12040730, PMID: 38674673 PMC11052236

[ref23] Parada Venegas D. de la Fuente M. K. Landskron G. González M. J. Quera R. Dijkstra G.. (2019). Short chain fatty acids (SCFAs)-mediated gut epithelial and immune regulation and its relevance for inflammatory bowel diseases. Front. Immunol. 10:277. doi: 10.3389/fimmu.2019.00277, PMID: 30915065 PMC6421268

[ref24] Penumutchu S. Korry B. J. Hewlett K. Belenky P. (2023). Fiber supplementation protects from antibiotic-induced gut microbiome dysbiosis by modulating gut redox potential. Nat. Commun. 14:5161. doi: 10.1038/s41467-023-40553-x, PMID: 37620319 PMC10449846

[ref25] Pirozzi C. Opallo N. Coretti L. Lama A. Annunziata C. Comella F.. (2023). Alkalihalobacillus clausii (formerly Bacillus clausii) spores lessen antibiotic-induced intestinal injury and reshape gut microbiota composition in mice. Biomed. Pharmacother. 163:114860. doi: 10.1016/j.biopha.2023.114860, PMID: 37196540

[ref26] Rivera-Chavez F. Zhang L. F. Faber F. Lopez C. A. Byndloss M. X. Olsan E. E.. (2016). Depletion of butyrate-producing Clostridia from the gut microbiota drives an aerobic luminal expansion of Salmonella. Cell Host Microbe 19, 443–454. doi: 10.1016/j.chom.2016.03.004, PMID: 27078066 PMC4832419

[ref27] Rose D. J. Poudel R. van Haute M. J. Yang Q. Wang L. Singh M.. (2021). Pulse processing affects gas production by gut bacteria during in vitro fecal fermentation. Food Res. Int. 147:110453. doi: 10.1016/j.foodres.2021.110453, PMID: 34399455

[ref28] Saez-Lara M. J. Gomez-Llorente C. Plaza-Diaz J. Gil A. (2015). The role of probiotic lactic acid bacteria and bifidobacteria in the prevention and treatment of inflammatory bowel disease and other related diseases: a systematic review of randomized human clinical trials. Biomed. Res. Int. 2015:505878. doi: 10.1155/2015/505878 25793197 PMC4352483

[ref29] Seo A. Y. Kim N. Oh D. H. (2013). Abdominal bloating: pathophysiology and treatment. J. Neurogastroenterol. Motil. 19, 433–453. doi: 10.5056/jnm.2013.19.4.433, PMID: 24199004 PMC3816178

[ref30] Sheng K. Xu Y. Kong X. Wang J. Zha X. Wang Y. (2021). Probiotic Bacillus cereus alleviates dextran sulfate sodium-induced colitis in mice through improvement of the intestinal barrier function, anti-inflammation, and gut microbiota modulation. J. Agric. Food Chem. 69, 14810–14823. doi: 10.1021/acs.jafc.1c03375, PMID: 34677958

[ref31] Slifer Z. M. Blikslager A. T. (2020). The integral role of tight junction proteins in the repair of injured intestinal epithelium. Int. J. Mol. Sci. 21:972. doi: 10.3390/ijms21030972, PMID: 32024112 PMC7036844

[ref32] Stofilova J. Kvaková M. Kamlárová A. Hijová E. Bertková I. Guľašová Z. (2022). Probiotic-based intervention in the treatment of ulcerative colitis: conventional and new approaches. Biomedicines 10:2236. doi: 10.3390/biomedicines10092236, PMID: 36140337 PMC9496552

[ref33] Sun Q. Ji Y. C. Wang Z. L. She X. He Y. Ai Q.. (2021). Sodium butyrate alleviates intestinal inflammation in mice with necrotizing enterocolitis. Mediat. Inflamm. 2021:6259381. doi: 10.1155/2021/6259381 PMC8526205 34675753

[ref34] Tan H. Chen X. Wang C. Song J. Xu J. Zhang Y.. (2023). Intestinal organoid technology and applications in probiotics. Crit. Rev. Food Sci. Nutr. 65, 1–15. doi: 10.1080/10408398.2023.2288887 38032232

[ref35] Tao Z. Wang Y. (2024). The health benefits of dietary short-chain fatty acids in metabolic diseases. Crit. Rev. Food Sci. Nutr. 65, 1–14. doi: 10.1080/10408398.2023.2297811 38189336

[ref36] Wang Q. Wang F. Zhou Y. Li X. Xu S. Jin Q.. (2024). Bacillus amyloliquefaciens SC06 relieving intestinal inflammation by modulating intestinal stem cells proliferation and differentiation via AhR/STAT3 pathway in LPS-challenged piglets. J. Agric. Food Chem. 72, 6096–6109. doi: 10.1021/acs.jafc.3c05956, PMID: 38484112

[ref37] Wang Y. Wu Y. Sailike J. Sun X. Abuduwaili N. Tuoliuhan H.. (2020). Fourteen composite probiotics alleviate type 2 diabetes through modulating gut microbiota and modifying M1/M2 phenotype macrophage in db/db mice. Pharmacol. Res. 161:105150. doi: 10.1016/j.phrs.2020.105150, PMID: 32818655

[ref38] Wu J. Chen N. Grau E. Johnson L. Liu Y. Li C.. (2024). Short chain fatty acids inhibit corneal inflammatory responses to TLR ligands via the ocular G-protein coupled receptor 43. Ocul. Surf. 32, 48–57. doi: 10.1016/j.jtos.2024.01.005, PMID: 38224777 PMC11056309

[ref39] Wu H. Xie S. Miao J. Li Y. Wang Z. Wang M.. (2020). Lactobacillus reuteri maintains intestinal epithelial regeneration and repairs damaged intestinal mucosa. Gut Microbes 11, 997–1014. doi: 10.1080/19490976.2020.1734423, PMID: 32138622 PMC7524370

[ref40] Xu J. Wang J. He Y. Chen R. Meng Q. (2024). L.acidophilus participates in intestinal inflammation induced by PM(2.5) through affecting the Treg/Th17 balance. Environ. Pollut. 341:122977. doi: 10.1016/j.envpol.2023.122977, PMID: 38006993

[ref41] Yao Y. Shang W. Bao L. Peng Z. Wu C. (2024). Epithelial-immune cell crosstalk for intestinal barrier homeostasis. Eur. J. Immunol. 54:e2350631. doi: 10.1002/eji.202350631, PMID: 38556632

[ref42] Yue Y. Wang Y. Xie Q. Lv X. Zhou L. Smith E. E.. (2023). Bifidobacterium bifidum E3 combined with Bifidobacterium longum subsp. infantis E4 improves LPS-induced intestinal injury by inhibiting the TLR4/NF-kappaB and MAPK signaling pathways in vivo. J. Agric. Food Chem. 71, 8915–8930. doi: 10.1021/acs.jafc.3c00421, PMID: 37255290

[ref43] Zegarra Ruiz D. F. Kim D. V. Norwood K. Saldana-Morales F. B. Kim M. Ng C.. (2022). Microbiota manipulation to increase macrophage IL-10 improves colitis and limits colitis-associated colorectal cancer. Gut Microbes 14:2119054. doi: 10.1080/19490976.2022.2119054, PMID: 36062329 PMC9450902

[ref44] Zeisel M. B. Dhawan P. Baumert T. F. (2019). Tight junction proteins in gastrointestinal and liver disease. Gut 68, 547–561. doi: 10.1136/gutjnl-2018-316906, PMID: 30297438 PMC6453741

[ref45] Zhang S. Zhao T. Wang Y. Mi J. Liu J. Fan X.. (2023). Intestinal microbiota regulates colonic inflammation in fluorosis mice by TLR/NF-kappaB pathway through short-chain fatty acids. Food Chem. Toxicol. 178:113866. doi: 10.1016/j.fct.2023.113866, PMID: 37269894

[ref46] Zhou M. Xu W. Wang J. Yan J. Shi Y. Zhang C.. (2018). Boosting mTOR-dependent autophagy via upstream TLR4-MyD88-MAPK signalling and downstream NF-kappaB pathway quenches intestinal inflammation and oxidative stress injury. EBioMedicine 35, 345–360. doi: 10.1016/j.ebiom.2018.08.035, PMID: 30170968 PMC6161481

[ref47] Zhou F. Zhang Q. Zheng X. Shi F. Ma K. Ji F.. (2024). Antiaging effects of human fecal transplants with different combinations of Bifidobacterium bifidum LTBB21J1 and Lactobacillus casei LTL1361 in d-galactose-induced mice. J. Agric. Food Chem. 72, 9818–9827. doi: 10.1021/acs.jafc.3c09815, PMID: 38647087

[ref48] Zmora N. Levy M. Pevsner-Fishcer M. Elinav E. (2017). Inflammasomes and intestinal inflammation. Mucosal Immunol. 10, 865–883. doi: 10.1038/mi.2017.19, PMID: 28401932

