# Supplementary figures and images for: Disclosing the benefits of multi-strain compounds and their health impact mechanisms utilizing intestinal biomimetic technology
Source: Front Microbiol. 2025 May 15;16:1550913. doi: 10.3389/fmicb.2025.1550913 (PMC12119514; doi:10.3389/fmicb.2025.1550913)

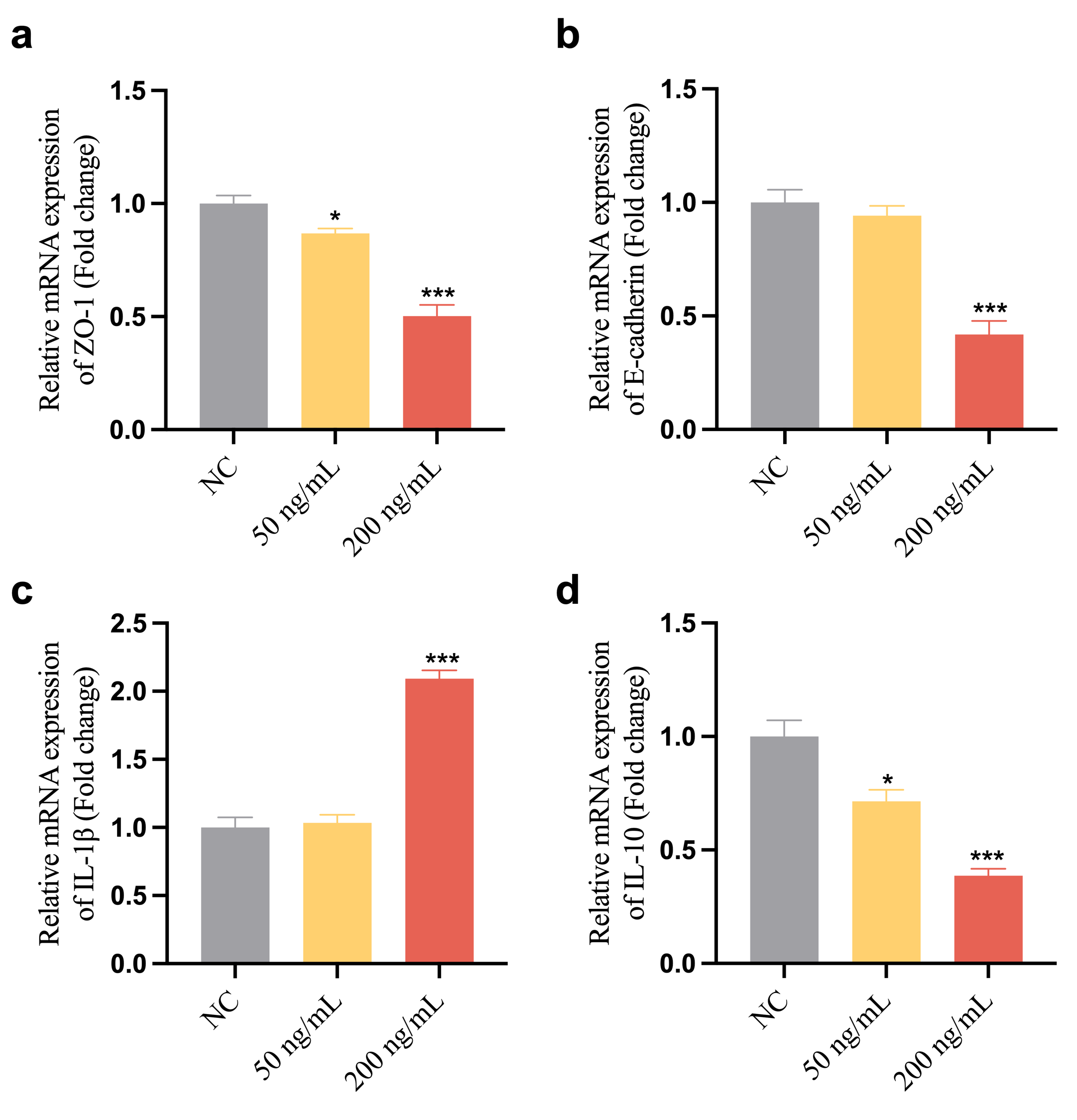

Supplement: SUPPLEMENTARY FIGURE S1 — Effect of different concentrations of IFN-γ on the expression of inflammatory cytokines and tight junction mRNA. (a) ZO-1; (b) E-cadherin; (c) IL-β; (d) IL-10. Data presented as means ± SEM, analyzed using one-way ANOVA. * p < 0.05, ** p < 0.01, *** p < 0.001. [file Image_1.TIF]
